# *Ureaplasma* Species Multiple Banded Antigen (MBA) Variation Is Associated with the Severity of Inflammation *In vivo* and *In vitro* in Human Placentae

**DOI:** 10.3389/fcimb.2017.00123

**Published:** 2017-04-13

**Authors:** Emma L. Sweeney, Suhas G. Kallapur, Simone Meawad, Tate Gisslen, Sally-Anne Stephenson, Alan H. Jobe, Christine L. Knox

**Affiliations:** ^1^Faculty of Health, School of Biomedical Sciences, Institute of Health and Biomedical Innovation, Queensland University of TechnologyBrisbane, QLD, Australia; ^2^Division of Neonatology, Cincinnati Children's Hospital Medical CentreCincinnati, OH, USA; ^3^Division of Neonatology, Department of Paediatrics, University of MinnesotaMinneapolis, MN, USA

**Keywords:** *Ureaplasma* species, preterm birth, chorioamnionitis, multiple banded antigen (MBA), virulence, host-microbe Interactions

## Abstract

**Background:** The multiple banded antigen (MBA), a surface-exposed lipoprotein, is a proposed virulence factor of *Ureaplasma* spp. We previously demonstrated that the number of *Ureaplasma parvum* MBA size variants in amniotic fluid was inversely proportional to the severity of chorioamnionitis in experimentally infected pregnant sheep. However, the effect of ureaplasma MBA size variation on inflammation in human pregnancies has not been reported.

**Methods:** Ureaplasmas isolated from the chorioamnion of pregnant women from a previous study (*n* = 42) were speciated/serotyped and MBA size variation was demonstrated by PCR and western blot. Results were correlated with the severity of chorioamnionitis and cord blood cytokines. *In vitro*, THP-1-derived macrophages were exposed to recombinant-MBA proteins of differing sizes and NF-κB activation and cytokine responses were determined.

**Results:** MBA size variation was identified in 21/32 (65.6%) clinical isolates (in 10 clinical isolates MBA size variation was unable to be determined). Any size variation (increase/decrease) of the MBA (regardless of *Ureaplasma* species or serovar) was associated with mild or absent chorioamnionitis (*P* = 0.023) and lower concentrations of cord blood cytokines IL-8 (*P* = 0.04) and G-CSF (*P* = 0.008). *In vitro*, recombinant-MBA variants elicited different cytokine responses and altered expression of NF-κB p65.

**Conclusion:** This study demonstrates that size variation of the ureaplasma MBA protein modulates the host immune response *in vivo* and *in vitro*.

## Introduction

The human *Ureaplasma* species (*U. parvum* and *U. urealyticum*) are prevalent colonizers of the lower genital tract and are known to colonize up to 80% of women and 50% of men (Cassell et al., 1993; Volgmann et al., 2005). Although these microorganisms were traditionally considered to be of low virulence (Volgmann et al., 2005), more recent evidence suggests that *Ureaplasma* spp. may be virulent pathogens of the female upper genital tract. The *Ureaplasma* spp. are the microorganisms isolated most frequently from the amniotic fluid and placentae of women (Hillier et al., 1988; Knox et al., 1997; Namba et al., 2010; Sweeney et al., 2016), either in the presence or absence of histological chorioamnionitis (Sweeney et al., 2016). These microorganisms have been associated with spontaneous abortion and miscarriage (Naessens et al., 1987, 1988), preterm birth (Hillier et al., 1988; DiGiulio et al., 2010), chorioamnionitis (Hillier et al., 1988; Namba et al., 2010; Sweeney et al., 2016), and preterm premature rupture of membranes (Jacobsson et al., 2009; DiGiulio et al., 2010). Despite the fact that *Ureaplasma* spp. have been isolated from up to 42% of pregnancies that end prematurely (Namba et al., 2010), the pathogenesis of ureaplasmas is not always clear; particularly as not all women who are infected with *Ureaplasma* spp. experience preterm birth or adverse pregnancy outcomes (Gerber et al., 2003). Several hypotheses have been proposed to explain this variation in pathology/outcomes of women infected with *Ureaplasma* spp. Researchers have suggested that there may be “virulent” *Ureaplasma* species or serovars (Naessens et al., 1988; Knox and Timms, 1998; De Francesco et al., 2009; Eun et al., 2013); however, links to a particular species/serovar with a disease state or adverse outcomes have not been consistent (Zheng et al., 1992). Others have suggested that virulence may not be limited to a single species or serovar of ureaplasmas, but instead may be associated with antigenic variation of the pathogen itself (Zheng et al., 1992, 1995, 1996; Dando et al., 2012).

The multiple banded antigen (MBA) is a major surface-exposed (Zheng et al., 1995; Shimizu et al., 2008), immunodominant antigen of *Ureaplasma* spp., which activates NF-κB and the production of cytokines by signaling *via* Toll-like receptors (TLRs) 1, 2, and 6 (Shimizu et al., 2008). More recently, Knox et al. (2010) demonstrated that the number of *U. parvum* MBA size variants present within the amniotic fluid of pregnant sheep (an ovine model of *Ureaplasma* spp. infection) was inversely proportional to the severity of inflammation within the chorioamnion. Sheep infected intraamniotically with the same strain and dose of *U. parvum* had divergent inflammatory responses within their chorioamnion: when >9 MBA size variants were present, there was little or no inflammation within the chorioamnion; by contrast, when there were five or fewer MBA size variants, severe inflammation of the chorioamnion was observed (Knox et al., 2010). Antigenic variation is a common feature of a wide range of pathogens; and to date the mechanisms known to govern *Ureaplasma* spp. antigenic variation include: (i) slipped strand mispairing and/or nucleotide insertions or deletions of simple repeating sequences and (ii) DNA rearrangements *via* site-specific recombination (Citti et al., 2010). While these studies have demonstrated a potential role for MBA size variation and variability in chorioamnionitis or pregnancy outcomes; it is currently unclear if MBA size variation can occur during infection of human placentae and if this variation results in similar outcomes to what we have previously reported in an ovine model.

Recently, our research group demonstrated that the *Ureaplasma* spp. were the most prevalent microorganisms (42/535; 7.8%) isolated from late preterm or term placentae and that their presence (but not the presence of other bacteria) was associated with chorioamnionitis. However, not all women whose placentae were infected/colonized with ureaplasmas had chorioamnionitis: in 15/42 (35.7%), no histological chorioamnionitis was observed (Sweeney et al., 2016). Using these same *Ureaplasma* spp. clinical isolates, we further investigated the role of MBA virulence in human ureaplasmas. We hypothesized size variation of the MBA protein (and gene) would occur in human *Ureaplasma* spp. clinical isolates, and that differences in the host immune response (*in vivo* and *in vitro*) would contribute to the severity of inflammation.

## Materials and methods

### Ethics statement

The use of human blood and tissues was approved by the review boards of the Good Samaritan Hospital (approval 09105-09-067) and Cincinnati Children's Hospital Medical Center (approval 2009-0236). All patients gave permission for the collection of their placentae upon delivery and for their medical records (demographic data and pregnancy/neonatal outcomes) to be recorded in a de-identified database, as described in our previous study (Shepard and Lunceford, 1976; Sweeney et al., 2016). Chorioamnion inflammation (chorioamnionitis) scores were performed by a pathologist, blinded to the microbiological findings, and the severity of chorioamnionitis was graded according to the guidelines set by Redline et al. (2003). All subjects gave written informed consent in accordance with the Declaration of Helsinki for their medical records to be recorded within a de-identified database. The work within this study was also submitted to the Human Research Ethics Committee (HREC) of the Queensland University of Technology and considered exempt from approval, as patient samples and data were de-identified prior to shipment/accessing of patient medical records. The QUT ethics committee also approved the production of recombinant proteins for use in *in vitro* experiments.

### *Ureaplasma* spp. clinical isolates

Tissue samples were excised from placentae and snap frozen prior to transport to Queensland University of Technology (QUT). At QUT, placental tissue samples were homogenized and ureaplasmas were cultured and ureaplasma DNA extracted as previously described (Sweeney et al., 2016). Clinical ureaplasma isolate cultures were also utilized for extraction of *Ureaplasma* proteins as previously described (Dando et al., 2012).

### Extraction of DNA from cultured ureaplasmas

Positive ureaplasma cultures from low passage (≤ 2 passages) isolates were centrifuged at 4,500 × g for 20 min (Allegra XR-15, Beckman Coulter, Australia) and nucleic acid was extracted from the resulting bacterial pellet using the QIAamp mini DNA extraction kit (Qiagen, Australia). All extracted DNA was stored at −20°C until required.

### Speciation and serotyping of *Ureaplasma* spp. clinical isolates

The upstream conserved region of the *Ureaplasma* spp. multiple banded antigen (*mba*) gene was performed as previously presented (Sweeney et al., 2016; Accession numbers: KY796009, KY796010, KY796011, KY796012, KY796013, KY796014, KY796015, KY796016, KY796017, KY796018, KY796019, KY796020, KY796021, KY796022, KY796023, KY796024, KY796025, KY796026, KY796027, KY796028, KY796029, KY796030, KY796031, KY796032, KY796033, KY796034, KY796035, KY796036, KY796037, KY796038, KY796039, KY796040, KY796041, KY796042, KY796043, KY796044, KY796045, KY796046, KY796047, KY796048, and KY796049) and the *mba* gene was used to serotype the *U. parvum* and *U. urealyticum* clinical isolates.

### PCR assays targeting the downstream repetitive region of the *mba* gene

The downstream repetitive region of *U. parvum* clinical isolates was amplified using previously published assays (Knox et al., 2010; Dando et al., 2012; Robinson et al., 2013). These primers amplified *U. parvum* serovars 1 and 6; or serovars 3 and 14 and revealed size variation within the *mba* gene.

### Western blotting of *Ureaplasma* spp. multiple banded antigen (MBA) protein

Cultures of each clinical isolate and *Ureaplasma* spp. ATCC strain were centrifuged at 4,500 × g for 20 min. The supernatant was then discarded and the pellet resuspended in 100 μL of sterile PBS. The suspensions were stored at −20°C prior to use. Extracted proteins (30 μg) were then used for western blot analysis, as previously described, to identify size variation in the MBA protein (Dando et al., 2012).

### Cord blood cytokine analysis

Cord blood was collected at the time of delivery from the umbilical vein using a sterile Viacord collection kit containing an anticoagulant, and the blood components separated by centrifugation. Concentrations of cytokines/chemokines within cord blood plasma were then determined using MILLIPLEX® MAP Human Cytokine/Chemokine magnetic bead panel (Millipore, USA). Concentrations of cytokines/chemokines were calculated from standard curves using recombinant proteins and the results were expressed in pg/mL.

### Production of recombinant MBA proteins from ureaplasmas

DNA from ATCC *U. parvum* serovar 6 and a selection of *U. parvum* serovar 6 clinical isolates (#27, #50, and #122 and #334B) were utilized to amplify the *mba* gene. The *mba* gene was selected from these clinical isolates (as they are representative the large variety of sizes observed within our *U. parvum* serovar 6 clinical isolates) in order to determine if the size of the *mba* gene (and the expressed MBA protein) alters the host immune response. All *mba* genes were selected from *U. parvum* serovar 6 isolates that were isolated from preterm placentae (<37 weeks of gestation). PCR assays were performed in 50 μL reactions, containing: 4 μL of DNA template, 50 μM dNTPs (ThermoFisher Scientific, Australia), 1.5 mM MgCl_2_ (ThermoFisher Scientific), 1 μM forward and reverse PCR primers (forward primer: ACATTAGGAGTTACC; reverse primer: TTATTTTCTAGCAGC; Sigma Aldrich) 2 units of *pfu* polymerase (Promega, Australia) and sterile DNAse/RNAse-free dH_2_0 (Gibco, Australia). PCR cycling was performed using the PTC-2000 thermocycler (Bio-Rad, Australia) and cycling consisted of: denaturation at 95°C for 5 min, followed by 35 cycles of denaturation at 95°C for 30 s, primer annealing at 55°C for 30 s and extension at 72°C for 2.5 min. PCR fragments were then purified using the PureLink PCR amplicon purification kit (ThermoFisher Scientific) and sequencing was performed (Life Technologies) to confirm the correct *mba* gene sequence.

The purified *mba* genes were ligated into the pRSET A plasmid (kindly provided by Professor Ken Beagley) and transformed into chemically competent DH5α *Escherichia coli*. Positive transformants for each *mba* gene/protein were then selected (a minimum of ten per gene/protein) for further analysis, to confirm the presence of the *mba* gene within the plasmid. Plasmids containing the correct insert were then transfected into BL21 *E. coli* and stored at −80°C for future use.

BL21 *E. coli* strains containing the *mba* genes were then used for large-scale protein production. This involved culturing *E. coli* strains until logarithmic growth was achieved, followed by induction of protein expression using isopropyl β-D-thiogalactoside (IPTG; Bioline, Australia). *E. coli* were then allowed to produce the protein of interest for 3 h, before being collected by centrifugation. The resulting cell pellet was resuspended in sterile phosphate-buffered saline (PBS). *E. coli* were then sonicated prior to protein purification.

MBA proteins were produced with a 6xHis-tag for use in ion metal chromatography using Talon® resin (ClonTech, Australia). MBA proteins were bound to the Talon® resin for 1 h before a series of washing steps with low concentrations (10 mM) of imidazole to remove any non-specific proteins. After washing, the protein of interest was eluted from the resin using 500 mM imidazole. Purified rMBA proteins were evaluated by western blot, using MBA-specific primary antibodies (kindly provided by Emeritus Dr Patricia Quinn, The Hospital for Sick Children, Toronto) to demonstrate the recombinant proteins were of the correct size. All proteins were confirmed to be free of contaminating LPS; any LPS detected was removed using a high capacity endotoxin removal kit (Life Technologies, Australia).

### *In vitro* stimulation of THP-1 macrophages using recombinant MBA proteins

THP-1 monocyte cells were grown in Roswell Park Memorial Institute (RPMI; ThermoFisher Scientific) media containing 10% fetal bovine serum, 1,000 U/mL benzylpenicillin and 0.05 mM β-mercaptoethanol. Cells were seeded into 48-well plates (Corning Life Sciences, Australia) at 1 × 10^5^ cells/well and supplemented with 10 ng/mL phorbol 12-myristate 13-acetate (PMA; Sigma Aldrich) to induce the differentiation of THP-1 monocytes to macrophages for 72 h (Park et al., 2007). After differentiation, the adherent cell monolayer was washed using sterile PBS and then exposed to 10 μg/mL of: ATCC *U. parvum* serovar 6 recombinant protein (~75 kDa), and *U. parvum* serovar 6 clinical isolate recombinant MBA proteins #27 (~37 kDa), #50 (~60 kDa), #122 (~70 kDa), and #334B (~75 kDa), suspended in RPMI culture media. Controls included no recombinant proteins (negative control), macrophages stimulated with 100 ng/mL *Escherichia coli* lipopolysaccharide (*E. coli* LPS; Sigma Aldrich) and macrophages exposed to 2 × 10^7^ colony forming units (CFU) of live or UV-inactivated (killed) *U. parvum* serovar 6. After 24 h, cell culture supernatant was collected for enzyme-linked immunosorbent assays (ELISA) for TNF-α, IL-1β IL-6, IL-8, IL-10, and G-CSF (ELISAkit.com, Australia). THP-1 cells exposed to each treatment group were also prepared for western blot analysis. Cells from each experimental group were scraped from the culture vessel (Sarstedt Pty. Ltd., Australia) and incubated in RIPA buffer (150 mM NaCl, 1% Triton X-100, 0.5% sodium deoxycholate, 0.1% sodium dodecyl sulfate, protease inhibitor cocktail, 50 mM Tris pH 8.0) at 4°C for 1 h, to extract the total cell protein. For SDS-PAGE electrophoresis, 60 μg of cell lysate from each sample was loaded into a gel for subsequent western blotting and transfer to a nitrocellulose membrane. The membrane was then stained with Ponceau S dye (Sigma Aldrich), in order to visualize the protein bands of the correct sizes, before the membrane was cut and blocked in 5% skim milk solution for 1 h. The membranes were then probed with human phosphorylated NF-κB p65 primary antibody (Abcam, Australia) or human β-actin primary antibody (Abcam) overnight at 4°C. The membranes were washed and probed with a rabbit anti-human IgG secondary antibody (Sigma Aldrich) for 1 h. Protein bands were visualized using 3′3′-diaminobenzidine (DAB) with metal enhancer (Sigma Aldrich). The developed membranes were then imaged using ChemiDoc MP imaging system (Bio-Rad) and densitometry analysis was performed using ImageJ software (NIH).

### Statistical analysis

All data are presented as the mean value, plus or minus the standard error of the mean (SEM). Data were analyzed using analysis of variance (ANOVA) and included adjustments for multiple comparisons. Statistical significance was accepted as *P* < 0.05.

## Results

### Speciation and serotyping of ureaplasma clinical isolates

The upstream conserved portion of the *mba* gene of 42 *Ureaplasma* spp. isolates were sequenced (Sweeney et al., 2016). The majority of these isolates were confirmed to be *U. parvum* (36/42; 85.7%) and only 6 (14.3%) *U. urealyticum* isolates were identified.

Of the 36 *U. parvum* clinical isolates, four were unable to be serotyped by sequencing; however, the remaining isolates were serotyped as *U. parvum* serovar 1 (11/32; 34.4%), serovar 3 (9/32; 28.1%), and serovar 6 (12/32; 37.5%). No *U. parvum* serovar 14 clinical isolates were detected. Of the *U. urealyticum* clinical isolates, only two of the six clinical isolates were able to be serotyped, and these were identified as *U. urealyticum* serovar 8 and serovar 10.

### Ureaplasma species and serovars: association with adverse pregnancy or neonatal outcomes

There were no differences in outcomes for neonates exposed to *U. parvum* or *U. urealyticum* (Table 1).

**Table 1:** **Maternal and neonatal demographic and outcome data for women whose placentae were infected with ***U. parvum or U. urealyticum*****.

	***U. parvum* (*n* = 36)**	***U. urealyticum* (*n* = 6)**	**Significance^a^**
**MATERNAL**			
Maternal age (years; mean, range)	24.7 ± 0.8 (17–32)	22.8 ± 2.0 (19–32)	0.046
Gravida (mean, range)	1.9 ± 0.2 (1–5)	3.2 ± 0.3 (2–4)	NS^b^
Parity (mean, range)	1.7 ± 0.2 (1–4)	2.3 ± 0.4 (1–4)	NS
At least one sign/symptom of infection^c^	4/36 (11.1%)	1/6 (16.7%)	NS
Preterm premature rupture of membranes (pPROM)	11/36 (30.6%)	4/6 (66.7%)	NS
**ETHNICITY**			
Caucasian	22/36 (61.1%)	1/6 (16.7%)	NS
African-American	12/36 (33.3%)	5/6 (83.3%)	NS
Asian	0/36 (0.0%)	0/6 (0.0%)	NS
More than one race	2/36 (5.6%)	0/6 (0.0%)	NS
**FETAL**			
Gestational age at delivery (weeks; mean, range)	35.9 ± 0.41 (32–41)	34.7 ± 0.61 (32–36)	NS
Birth weight (grams; mean, range)	2,550 ± 102.47 (1,380–3,873)	2674.17 ± 148.17 (2,290–3,330)	NS
Placental weight (grams; mean, range)	456.0 ± 22.9 (260–710.7)	400.4 ± 14.3 (374–461)	NS
Histologic Chorioamnionitis^d^	22/36 (61.1%)	5/6 (83.3%)	NS
Maternal Stage	1.5 ± 0.1 (1–3)	1.6 ± 0.2 (1–2)	NS
Fetal Stage	2.0 ± 0.2 (1–3)	3.0 ± 0.0^4^	NS
Continuous positive airway pressure (CPAP)	6/36 (16.7%)	2/6 (33.3%)	NS
Required oxygen support for > 6 h	4/36 (11.1%)	3/6 (50.0%)	NS
Diagnosed with respiratory distress syndrome (RDS)	5/36 (13.9%)	2/6 (33.3%)	NS
Length of stay in hospital (days; mean, range)	6 ± 1.2 (1–28)	5.5 ± 1.7 (2–13)	NS

a *Statistical significance determined by analysis of variance (ANOVA)*.

b *NS—not statistically significant*.

c *Defined as maternal temperature of >38°C, uterine or abdominal tenderness, foul-smelling vaginal discharge, maternal tachycardia (heart rate, >120 beats/min), or fetal tachycardia (heart rate, >160 beats/min)*.

d *Assessed using tissue sections from each placenta according to criteria set out in Redline et al. (2003). Maternal and fetal grades of chorioamnionitis are listed as median and range*.

We also compared the outcomes of mothers and their infants exposed to the most common serovars within our study: *U*. *parvum* serovars 1, 3, and 6. Women whose placentae were infected with *U. parvum* serovar 3 (*n* = 9) were younger (21.4 ± 0.9 years) than women in whom *U. parvum* serovars 1 (*n* = 11) or 6 (*n* = 12) were identified (25.6 ± 1.3 and 26.0 ± 1.4 years, respectively; *P* = 0.024) (Table 2). There were no other differences between these three groups of women, nor were there any differences in the incidence of adverse neonatal outcomes, including the prevalence of histological chorioamnionitis.

**Table 2:** **Maternal and neonatal demographic/outcome data for women whose placentae were infected with ***U. parvum*** serovars 1, 3, or 6**.

	**Serovar 1 (*n* = 11)**	**Serovar 3 (*n* = 9)**	**Serovar 6 (*n* = 12)**	**Significance^a^**
**MATERNAL**				
Maternal age (years; mean, range)	25.6 ± 1.3 (18–32)	21.4 ± 0.9 (17–26)	26.0 ± 1.4 (19–32)	0.024
Gravida (mean, range)	2.0 ± 0.3 (1–4)	1.7 ± 0.4 (1–4)	1.9 ± 0.4 (1–5)	NS^b^
Parity (mean, range)	1.9 ± 0.3 (1–4)	1.4 ± 0.2 (1–2)	1.8 ± 0.3 (1–4)	NS
At least one sign/symptom of infection^c^	2/11 (18.2%)	1/9 (11.1%)	0/12 (0.0%)	NS
Preterm premature rupture of membranes (pPROM)	2/11 (18.2%)	3/9 (33.3%)	3/12 (25.0%)	NS
**ETHNICITY**				
Caucasian	9/11 (81.8%)	7/9 (77.8%)	6/12 (50.0%)	NS
African-American	2/11 (18.2%)	2/9 (22.2%)	5/12 (41.7%)	NS
Asian	0/11 (0.0%)	0/9 (0.0%)	0/12 (0.0%)	NS
More than one race	0/11 (0.0%)	0/9 (0.0%)	1/12 (8.3%)	NS
**FETAL**				
Gestational age at delivery (weeks; mean, range)	36.5 ± 0.9 (33–41)	35.9 ± 0.7 (33–39)	35.3 ± 0.7 (32–40)	NS
Birth weight (grams; mean, range)	1,975 ± 174.1 (1,975–3,873)	2650.7 ± 219.5 (1,525–3,855)	2370.8 ± 186.63 (1,380–3,825)	NS
Placental weight (grams; mean, range)	524.2 ± 38.7 (270–635)	482.4 ± 48.5 (279–710.7)	395.8 ± 31.7 (260–655)	NS
Histologic Chorioamnionitis^d^	7/11 (63.6%)	5/9 (55.5%)	7/12 (58.3%)	NS
Maternal Stage	1.6 ± 0.2 (1–2)	1.25 ± 0.1 (1–2)	1.6 ± 0.3 (1–3)	NS
Fetal Stage	2.5 ± 0.2 (2–3)	2 ± 0.3 (1–3)	1.7 ± 0.3 (1–3)	NS
Continuous positive airway pressure (CPAP)	1/10 (10%)	2/9 (22.2%)	3/12 (25.0%)	NS
Required oxygen support for > 6 h	1/10 (10%)	2/9 (22.2%)	2/12 (16.7%)	NS
Diagnosed with respiratory distress syndrome (RDS)	1/10 (10%)	2/9 (22.2%)	2/12 (16.7%)	NS
Length of stay in hospital (days; mean, range)	7.6 ± 2.7 (1–28)	5.0 ± 2.1 (1–16)	6.9 ± 1.8 (2–17)	NS

a *Statistical significance determined by analysis of variance (ANOVA)*.

b *NS—not statistically significant*.

c *Defined as maternal temperature of >38°C, uterine or abdominal tenderness, foul-smelling vaginal discharge, maternal tachycardia (heart rate, >120 beats/min), or fetal tachycardia (heart rate, >160 beats/min)*.

d *Assessed using tissue sections from each placenta according to criteria set out in Redline et al. (2003). Maternal and fetal grades of chorioamnionitis are listed as median and range*.

### PCR and western blot targeting the MBA protein and *mba* gene

**Figure 1:**
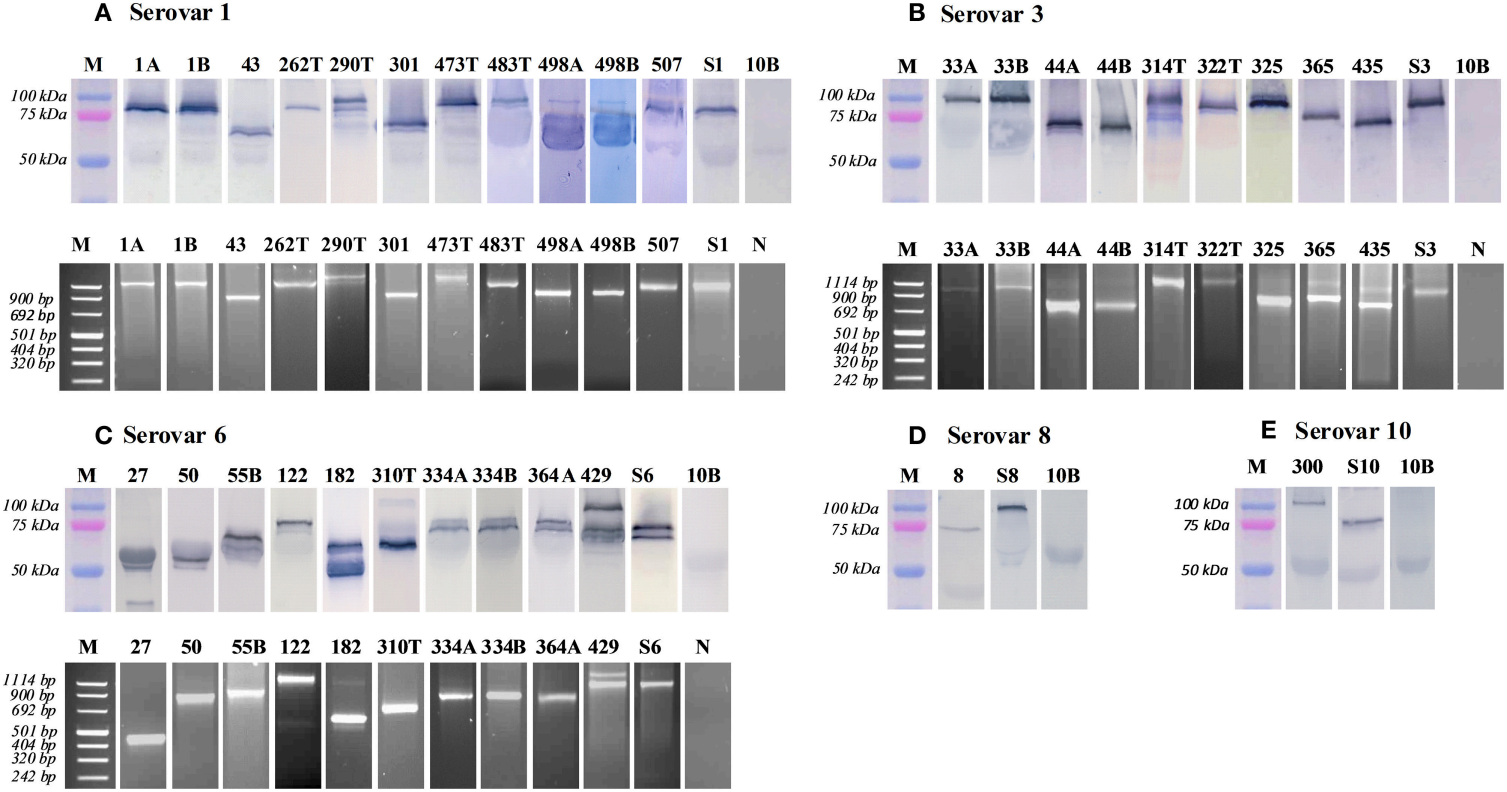
**Variation of the MBA protein and ***mba*** gene was detected by western blot and PCR**. MBA/*mba* size variation was characterized by comparing the protein and PCR amplicons to the ATCC strain serovar control and a molecular weight marker bands. Images were cropped and displayed at their correct molecular weight and sorted according to their serovar. M, protein marker; 10B, 10B media control; N, negative control; S, serovar 1, 3, 6, 8, 10—ATCC positive controls; A/B, twin pregnancies; T, term pregnancy (where all others are late preterm: 32–36 weeks of gestation). **(A)** serovar 1. **(B)** serovar 3. **(C)** serovar 6. **(D)** serovar 8. **(E)** serovar 10.

The MBA protein and *mba* gene of ureaplasma clinical isolates were compared to American Type Culture Collection (ATCC) strain controls (serovar 1, 3, 6, 8, and 10, which served as positive controls for the size/expression of the MBA/*mba*). Numerous MBA/*mba* size variants were detected within low-passage (≤ passage 2) clinical isolates (Figure 1). For some ureaplasma clinical isolates, there was no identifiable MBA/*mba* size variation, i.e., the MBA protein and *mba* gene were the same size as the antigens/genes of the ATCC strain serovars (Serovar 1 isolates: 1A, 1B, 262T, 507; Serovar 3 isolates: 33A, 33B, 322T, 325; and Serovar 6 isolates: 334A, 334B, 364A); for other clinical isolates, variation in the size of their MBA protein and *mba* gene band(s) were observed. These clinical isolates demonstrated either a “single MBA/*mba* size variant,” which was considered to be an individual protein/gene band that differed in size when compared to the corresponding ATCC strain controls (Serovar 1 isolates: 43, 301, 473T, 483T, 498A, 498B; Serovar 3 isolates: 44A, 44B, 314T, 365, 435; Serovar 6 isolates: 27, 50, 55B, 122, and 310T; Serovar 8 isolate: 8; and Serovar 10 isolate 300); or in some cases, “multiple MBA/*mba* variant” bands were seen, where more than one MBA/*mba* band was visualized by PCR or western blot (Serovar 1 isolates: 290T; Serovar 6 isolates: 182, 429) (Figure 1). We did not see any difference in the propensity for these *Ureaplasma* isolates to vary the size of their MBA/*mba*, according to the species or serovar identified (Table 3).

**Table 3:** **MBA size variation in ***U. parvum*** serovars**.

	**Serovar 1 (*n* = 11)**	**Serovar 3 (*n* = 9)**	**Serovar 6 (*n* = 10)^a^**	**Significance**
No MBA size variants	4/11 (36.4%)	4/9 (44.4%)	3/10 (30.0%)	NS
Single MBA size variant	6/11 (54.5%)	5/9 (55.6%)	5/10 (50.0%)	NS
Multiple MBA size variants	1/11 (9.1%)	0/9 (0.0%)	2/10 (20.0%)	NS

*We identified no differences in the ability of U. parvum serovars to vary the size of their MBA/mba*.

a *No MBA was detected for 2 of the U. parvum serovar 6 clinical isolates (n = 12 in total)*.

For some clinical isolates, *n* = 2 *U. parvum* serovar 6 isolates and *n* = 8 *U. urealyticum* isolates the MBA protein and *mba* gene were not detected or visualized. Therefore, we were unable to determine if MBA/*mba* size variation occurred in these clinical isolates.

### MBA/*mba* size variation was associated with altered immune responses *In vivo*

**Figure 2:**
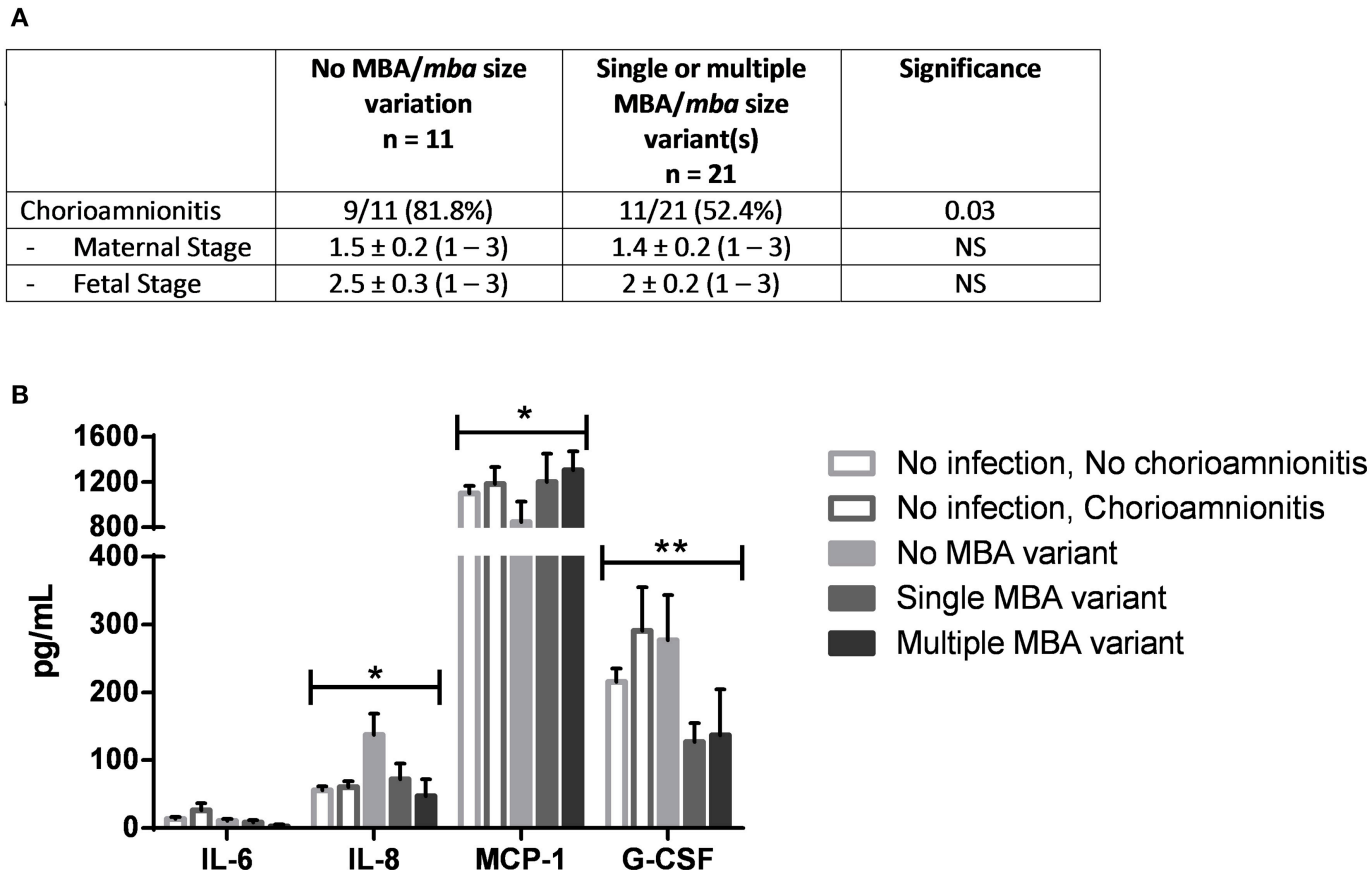
**MBA/***mba*** size variation was associated with the incidence and severity of histological chorioamnionitis. (A)** Histological chorioamnionitis was more prevalent in placentae infected with *Ureaplasma* spp. that did not vary their MBA/*mba*, when compared to placentae infected with *Ureaplasma* spp. that expressed a single or multiple MBA/*mba* size variants **(B)** MBA/*mba* size variation was also associated with altered levels of cord blood cytokines *in vivo*. ^*^P < 0.05, ^**^P < 0.01.

The maternal demographic data of ureaplasma-infected women in which MBA/*mba* variation was identified was compared to those women in whom no MBA/*mba* size variation was seen. No differences were observed (data not shown). However, a major finding of this study was that *Ureaplasma* spp. MBA/*mba* size variation was associated with differences in the incidence of histological chorioamnionitis as graded by a US pathologist (Figure 2A). Cord blood cytokines from women in which no microorganisms were detected (by culture and PCR) were compared to the cord blood cytokine profiles from women whose placentae were found to be infected with *Ureaplasma* spp. Placentae which harbored ureaplasmas with no MBA/*mba* size variation demonstrated a higher prevalence of histological chorioamnionitis (9/11; 81.8%), when compared to placentae in which the ureaplasmas present expressed either a single or multiple MBA/*mba* size variant(s) (10/21; 47.6% *p* = 0.03). No differences in the severity of inflammation within the maternal and fetal sides of these membranes were observed (Figure 2A). There were no other differences in pregnancy or neonatal outcomes associated with ureaplasma MBA/*mba* size variation.

Cord blood samples collected at the time of delivery were also tested for inflammatory markers (cytokines, chemokines, and growth factors including IL-1β, IL-6, IL-8, MCP-1, and G-CSF). *In vivo*, when ureaplasma MBA/*mba* size variation occurred within infected placentae, lower concentrations of IL-8 (67.7 pg/mL) and G-CSF (128.7 pg/mL) were detected, when compared to the concentrations of these same cytokines in placentae which were infected with *Ureaplasma* spp. but demonstrated no MBA/*mba* size variation (IL-8: 137.7 pg/mL, G-CSF: 277.0 pg/mL; *P* = 0.044, *P* = 0.008, respectively; Figure 2B).

In contrast, levels of MCP-1 in cord blood were significantly elevated when ureaplasmas which demonstrated MBA/*mba* size variation were isolated from placentae, when compared to the cord blood collected from those placentae which were infected with ureaplasmas that showed no variation in the size of their MBA/*mba* (*P* = 0.048).

### MBA size variation was also associated with altered immune responses *In vitro*

To further investigate the role of MBA size variation and the host response, we challenged differentiated human THP-1 (macrophages) with recombinant MBA proteins of differing sizes, using *Escherichia coli* lipopolysaccharide (LPS) as a positive control. These recombinant MBA proteins were produced from *U. parvum* serovar 6 ATCC strain (control), and from *U. parvum* serovar 6 clinical isolates (isolates: #27, #50, #122, and #334B; see Figure 1).

**Figure 3:**
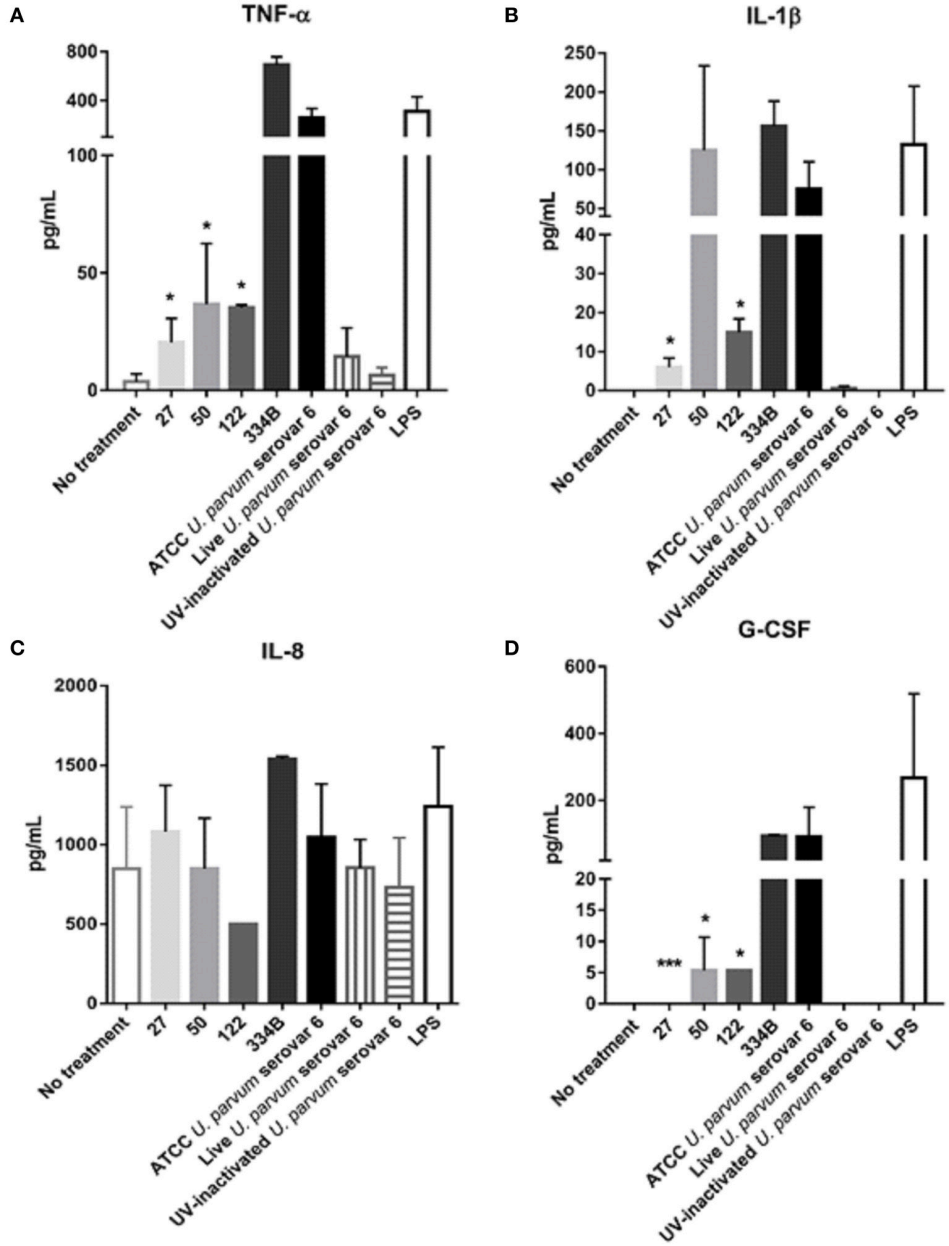
**Recombinant MBA proteins of differing sizes elicit varying immune responses in THP-1 macrophages ***in vitro*****. Assays were conducted on cell culture supernatant taken from differentiated THP-1 (macrophages) exposed to recombinant proteins, or controls, for 24 h. ELISA assays for **(A)** TNF-a, **(B)** IL-lB, **(C)** IL-8, and **(D)** G-CSF expressed in picograms per milliliter of culture supernatant. ^*^P < 0.05, ^**^P < 0.01, ^***^P < 0.001.

When THP-1 macrophages were exposed to the *E. coli* LPS control, robust immune responses were detected for the cytokines TNF-α, IL-1β, IL-8, and G-CSF. *In vitro*, the ATCC *U. parvum* recombinant MBA (rMBA; ~75 kDa) protein elicited the strongest immune response of all rMBA proteins tested; and stimulated the production of TNF-α, IL-1β, IL-8, and G-CSF (Figure 3). Similarly, the equivalent size rMBA protein of isolate #334B (~75 kDa) elicited similar immune responses. In contrast, recombinant proteins that were smaller in size (compared to ATCC *U. parvum* serovar 6 and the rMBA #334B) elicited diminished cytokine responses. The rMBA #50 protein (~60 kDa) elicited lower concentrations of TNF-α (36.6 ± 25.9 pg/mL; *P* = 0.024) and G-CSF (5.3 ± 1.3 pg/mL; *P* = 0.044), when compared to the concentrations seen in response to the rMBA of ATCC *U. parvum* serovar 6.; while the rMBA #122 (~70 kDa) elicited lower levels of TNF-α (35.2 ± 1.1 pg/mL; *P* = 0.020), IL-1β (14.9 ± 2.1 pg/mL; *P* = 0.045), and G-CSF (5.3 ± 1.7 pg/mL; *P* = 0.039). The smallest rMBA #27 (~37 kDa) elicited the production of cytokines at similar levels to that of our negative (no treatment) controls (TNF-α: *P* = 0.002, IL-1β: *P* = 0.033, G-CSF: *P* < 0.001; Figure 3), with the exception of IL-8. Interestingly, regardless of the challenge (LPS, *U. parvum* controls, no treatment and rMBA proteins) there were no differences in the concentrations of IL-8. Similarly, no significant differences were seen in IL-6 and IL-10 cytokine levels in cell culture supernatants (data not shown).

**Figure 4:**
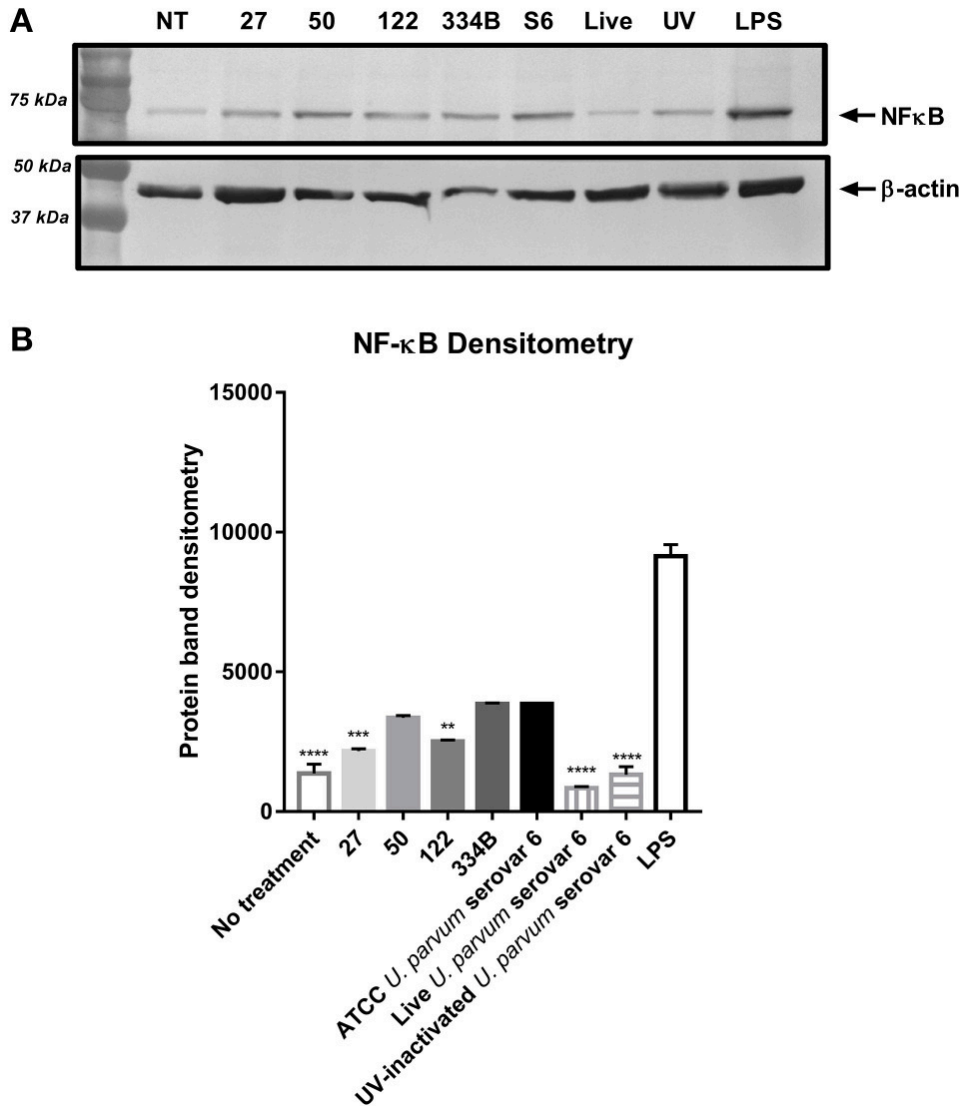
**NF -KB p65 expression alters upon exposure to different size recombinant MBA proteins ***in vitro***. (A)** NF-KB p 65 expression is altered upon exposure to recombinant MBA proteins of differing sizes, when normalized to host (β-actin) controls. **(B)** Densitometry analyses from *n* = 3 experiments, performed in triplicate. ^*^P < 0.05, ^**^P < 0.01, ^***^P < 0.001.

Since altered cytokine responses were observed when macrophages were exposed to different rMBA protein size variants, we further investigated the activation/expression of NF-κB p65, the protein complex that controls transcription of DNA and cytokine production, in cells exposed to the different sized rMBA proteins. Western blot and densitometry analyses revealed that the expression of NF-κB was highest for cell lysates exposed to LPS, ATCC *U. parvum* serovar 6 and rMBA #334B (Figures 4A,B). By contrast, diminished expression of NF-κB p65 was observed in cell lysates after exposure to rMBAs #27 and #122, relative to host (β-actin) controls (Figure 4).

## Discussion

The pathogenic role of *Ureaplasma* spp. remains controversial, as ureaplasma infection within placentae is not always associated with inflammation and adverse pregnancy outcomes (Gerber et al., 2003). Furthermore, the host immune response which influences the development of pathology/disease has not been well-studied during *Ureaplasma* spp. infections. A previous study by our group demonstrated that *Ureaplasma* spp. were the most prevalent bacteria detected within the chorioamnion of women (7.8%) who delivered late preterm or at term, but not all women infected with ureaplasmas developed histological chorioamnionitis (Sweeney et al., 2016). In this current study, we investigated the factors previously implicated in adverse pregnancy outcomes and inflammation during *Ureaplasma* spp. infection.

Similar to our previous study, in which *U. parvum* was the most frequently isolated bacterial species (86%) (Sweeney et al., 2016), in this current study we identified that *U. parvum* serovars 1, 3, and 6 were the most common ureaplasma serovars detected within infected placentae. However, we found that regardless of the *Ureaplasma* species or serovar isolated within the chorioamnion, the incidence of chorioamnionitis and other adverse pregnancy/neonatal outcomes did not differ (Tables 1, 2). Previous studies have suggested the existence of “virulent” *Ureaplasma* species or serovars (Naessens et al., 1988; Knox and Timms, 1998; Knox et al., 2003; Dando et al., 2012; Sweeney et al., 2016); however, ureaplasmas may not be intrinsically “virulent” or “avirulent,” rather, that other factors may instead contribute to adverse outcomes during ureaplasma infections. Furthermore, our findings are consistent with previous studies that have shown *Ureaplasma* spp. to be associated with chorioamnionitis (Hillier et al., 1988; Cassell et al., 1993; Namba et al., 2010), but ureaplasmas may also be detected within the placentae of women with no evidence of chorioamnionitis and the pregnancy may continue until term delivery, as was also reported by Gerber et al. (2003).

The most significant finding of this study was that *Ureaplasma* spp. isolated from the placentae of human pregnancies demonstrated differences in the size of their MBA protein and *mba* gene. Some of the clinical ureaplasma isolates obtained from chorioamnion tissue expressed no MBA size variation (and their proteins were the same size as our ATCC strain controls), while other clinical isolates expressed a single or multiple MBA size variants (as evidenced by single or multiple MBA bands which differed in size, when compared to the control serovar ATCC strains). While we saw no differences in the propensity of the different *Ureaplasma* species or serovars to vary the size of their MBA/*mba* (Table 3), our study demonstrated that a lack of MBA/*mba* size variation *in vivo* was associated with a significantly higher incidence of histological chorioamnionitis (Figure 2A) and elevated levels of the cord blood cytokines IL-8 and G-CSF (Figure 2B). By contrast, when MBA/*mba* size variation occurred, this was associated with a significant (~30%) reduction in the incidence of histological chorioamnionitis and significantly lower levels of the cord blood cytokines IL-8 and G-CSF (*P* = 0.04 and *P* = 0.008; Figure 2B).

Previous studies have indicated that the severity of chorioamnionitis varied, depending on the numbers of *Ureaplasma* spp. present within placental tissue (Jacobsson et al., 2009; Kasper et al., 2010; Kacerovsky et al., 2011); however, within our previous study of *n* = 535 placentae we did not find an association between the numbers of ureaplasmas present within the chorioamnion and the severity of chorioamnionitis (Sweeney et al., 2016). Instead, the results of this current study support the proposal that the severity of inflammation is associated with the degree of MBA/*mba* size variation.

We further investigated the role of MBA/*mba* size variation using an *in vitro* cell culture model: differentiated THP-1 (macrophage) cells were stimulated with rMBA *U. parvum* serovar 6 proteins of differing sizes. Of the rMBA proteins tested, the ATCC *U. parvum* serovar 6 protein elicited the greatest cytokine response, followed closely by the rMBA protein #334B, which was equivalent in size to that of the ATCC strain. In contrast, rMBA serovar 6 proteins that differed in size to the ATCC *U. parvum* serovar 6 strain and #334B proteins elicited lower concentrations of cytokines TNF-α, IL-1β, and G-CSF. For the rMBAs #27 and #122 significantly lower concentrations of IL-1β were also elicited when these proteins were exposed to THP-1 cells (Figures 3A–D). However, no differences were observed in the concentrations of IL-6 or IL-10 in the cell culture supernatant (data not shown). Of great interest, the smallest recombinant MBA protein #27 elicited minimal cytokine production and these concentrations of cytokines were similar to those in response to the negative (no treatment) control, indicating that the macrophages within this study may be unable to recognize this protein. These findings were confirmed by the results of densitometry analyses of NF-κB p65, the protein complex that controls transcription of DNA and cytokine production (Rahman and McFadden, 2011). Altered expression of NF-κB p65 was demonstrated in cell lysates exposed to rMBA proteins that were different in size, when compared to ATCC *U. parvum* serovar 6 and #334B recombinant proteins. NF-κB p65 expression was decreased for recombinant proteins #27 and #122 (Figures 4A,B), and this corresponded directly to the diminished cytokine levels seen *in vitro* (Figure 3).

It was first reported in 2008 that the *Ureaplasma* spp. surface-exposed lipoproteins, predominantly the MBA, induced an inflammatory response that resulted in the induction of NF-κB though TLRs 1, 2, and 6; and this study further proposed that size variation of the MBA may affect the stimulatory activity of the ureaplasma MBA and its ability to interact with TLRs (Shimizu et al., 2008). Others have reported the cytokine responses elicited when: (i) adult human monocytes and term and preterm neonatal cord blood were exposed to low (10^3^ color changing units [CCU]) or high doses (10^6^ CCU) of *U. parvum* serovars 3 (Manimtim et al., 2001), (ii) human monocytes (THP-1 cells) were treated with heat-killed *U. urealyticum* (Li et al., 2000), (iii) THP-1 macrophages were exposed to surface lipoproteins of *Ureaplasma urealyticum* (Peltier et al., 2007), and (iv) human amniotic epithelial cells were exposed to *Ureaplasma* spp. serovars 2, 3, and 14 (1 × 10^8^ bacteria/mL) (Triantafilou et al., 2013). In each of these *in vitro* experiments, the production of TNF-α was elevated in response to the ureaplasmas/antigens presented. However, the production of other cytokines (IL-1β, IL-8, IL-6, and IL-10) varied depending on the cell type, the bacterial or antigenic load, and the presence of LPS or steroids. A major strength of our current study was the correlation of immune responses in differentiated THP-1 (macrophages) to the same ureaplasma serovar (live or UV-inactivated *U. parvum* serovar 6) and rMBA proteins of differing sizes that were synthesized from low-passage *U. parvum* serovar 6 clinical isolates originally isolated from human placentae. These experiments confirm that rMBA proteins of differing sizes elicit varying concentrations of cytokines (IL-1β, IL-8, MCP-1, and G-CSF), depending on the size of the MBA antigens expressed by the ureaplasmas *in vitro* or *in vivo*.

Antigenic variation is an important mechanism used by microorganisms to mediate their interaction with the host and/or environment and this variation is thought to be an essential strategy employed by microorganisms to assist in pathogen survival, particularly in the presence of a host immune response (Citti et al., 2010). Antigen variation is not a unique trait of *Ureaplasma* spp., many microbes possess the ability to vary their surface exposed antigens (Citti et al., 2010; Foley, 2015); however, MBA/*mba* size variation has been reported previously in a sheep model of intraamniotic ureaplasma infection (Knox et al., 2010; Dando et al., 2012; Robinson et al., 2013). To the best of our knowledge, this current study is the first to identify variation of the *Ureaplasma* spp. MBA/*mba* in human placentae, and to demonstrate that this variation is associated with the incidence and severity of chorioamnionitis during pregnancy. Furthermore, this study is also the first to demonstrate that levels of cord blood cytokines differ when the placenta of pregnant women infected with ureaplasmas express different MBA/*mba* size variants.

Previous studies have investigated the role of size variation of the MBA/*mba*. Initial studies demonstrated the ability of *Ureaplasma* spp. isolated from neonates to vary their surface-exposed MBA/*mba* (Zheng et al., 1992) and that variation of the MBA/*mba* correlated with the number of repeating units in the downstream (surface-exposed) portion of the protein and gene (Zheng et al., 1995). These authors suggested that further knowledge of MBA size variation “would be requisite to understanding the role that these antigens and their associated size variation may play in the success or failure of these organisms as pathogens” (Zheng et al., 1995). Further studies by this same group utilized antibody-reactive peptide scanning and showed that differences in the numbers of repeating units within the MBA were associated with altered recognition of the MBA by sera which contained anti-ureaplasma antibodies (Zheng et al., 1996). In this previous study an interesting trend was observed; the ability of the monoclonal antibodies to bind to the MBA increased with the number of repeating units present (Zheng et al., 1996). This finding correlates with our data, which demonstrated that MBA size variation resulted in altered immune responses, both *in vivo* and *in vitro* and suggests that MBA size variation may be a mechanism by which ureaplasmas can evade either innate or adaptive immune responses.

Zimmerman et al. (2009, 2013) in *in vitro* experiments demonstrated inversion events that occurred between the *mba* and other regions with the genome of *U. parvum* serovars 3. These events were confirmed on both the genomic and protein level. They concluded that these DNA inversion events are dynamic and result in the high-frequency, broad spectrum, antigenic variation of these pathogens. While in these studies, DNA inversion events were not characterized *in vivo*, studies by Dando et al. (2012) identified ovine anti-ureaplasma IgG antibodies within maternal and fetal serum that were collected after sheep were infected intraamniotically with the same *U. parvum* low passage clinical isolate. This study revealed that the anti-ureaplasmal antibodies from these animals reacted with more than one ureaplasma MBA size variant, and that the IgG reactivity differed between animals (Dando et al., 2012). This study confirmed that MBA/*mba* size variation occurred *in vivo* and we and others (Zheng et al., 1992, 1995, 1996; Monecke et al., 2003; Zimmerman et al., 2009, 2011, 2013) propose that this variation is an important immune evasion mechanism for *Ureaplasma* spp. Similar antigenic variation leading to immune evasion has been noted in several other well-studied organisms, including *Plasmodium falciparum, Mycoplasma pulmonis*, and the human immunodeficiency virus (Lipsitch and O'Hagan, 2007; Citti et al., 2010). Further studies are required to understand the immune pressures which may trigger MBA/*mba* size variation *in vivo* and *in vitro* and to determine how these changes of the organism facilitates immune evasion.

In summary, this is the first study to demonstrate MBA/*mba* size variation within ureaplasmas isolated from the human chorioamnion. Our data suggest that *Ureaplasma* species or serovars are not intrinsically “virulent” or “avirulent,” but instead that variation of the ureaplasma MBA protein may play an important role in modulating the immune response: when MBA/*mba* size variation occurred *in vivo* there was a decrease in the incidence of chorioamnionitis and lower levels of the cord blood cytokines IL-8 and G-CSF. This hypothesis was also supported by our *in vitro* findings that recombinant MBA proteins of different sizes elicited altered cytokine responses and augmented expression of NF-κB p65 in macrophages. While variation in the size of the MBA/*mba* did not always result in an abolished immune response *in vitro* and *in vivo*, the responses to these variants were often diminished and this is consistent with an immune evasion event. The ability of these microorganisms to alter/modulate the host immune response may be a contributing factor to the virulence of the *Ureaplasma* spp. in establishing chronic, asymptomatic infections *in utero*, and highlights the need for future studies of these microorganisms as underestimated pathogens of pregnancy.

## Author contributions

Conceived and designed the experiments: ES, SK, TG, SS, AJ, and CK. Performed the experiments/generated data within the manuscript: ES, SK, SM, TG, AJ, and CK. Analyzed/Interpreted the data within the manuscript: ES, SK, SM, TG, SS, AJ, and CK. Contributed to the writing/revising of the manuscript: ES, SK, SM, TG, SS, AJ, and CK.

### Conflict of interest statement

The authors declare that the research was conducted in the absence of any commercial or financial relationships that could be construed as a potential conflict of interest.
